# Identification and functional characterisation of 5-HT4 receptor in sea cucumber *Apostichopus japonicus* (Selenka)

**DOI:** 10.1038/srep40247

**Published:** 2017-01-06

**Authors:** Tianming Wang, Zhen Yang, Naiming Zhou, Lina Sun, Zhenming Lv, Changwen Wu

**Affiliations:** 1 National Engineering Research Center of Marine Facilities Aquaculture, Marine Science College, Zhejiang Ocean University, Zhoushan, Zhejiang 316022, People’s Republic of China; 2 Institute of Biochemistry, College of LifeSciences, Zijingang Campus, Zhejiang University, Hangzhou, Zhejiang 310058, People’s Republic of China; 3 Key Laboratory of Marine Ecology and Environmental Sciences, Institute of Oceanology, Chinese Academy of Sciences, Qingdao, Shandong 266071, People’s Republic of China

## Abstract

Serotonin (5-HT) is an important neurotransmitter and neuromodulator that controls a variety of sensory and motor functions through 5-HT receptors (5-HTRs). The 5-HT4R subfamily is linked to Gs proteins, which activate adenylyl cyclases (ACs), and is involved in many responses in peripheral organs. In this study, the 5-HT4R from *Apostichopus japonicus (Aj*5-HT4R) was identified and characterised. The cloned full-length *Aj*5-HT4R cDNA is 1,544 bp long and contains an open reading frame 1,011 bp in length encoding 336 amino acid proteins. Bioinformatics analysis of the *Aj*5-HT4R protein indicated this receptor was a member of class A G protein coupled receptor (GPCR) family. Further experiments using *Aj*5-HT4R-transfected HEK293 cells demonstrated that treatment with 5-HT triggered a significant increase in intracellular cAMP level in a dose-dependent manner and induced a rapid internalisation of *Aj*5-HT4R fused with enhanced green fluorescent protein (*Aj*5-HT4R-EGFP) from the cell surface into the cytoplasm. In addition, the transcriptional profiles of *Aj5-HT4R* in aestivating *A. japonicas* and phosphofructokinase (*AjPFK*) in 5-HT administrated *A. japonicus* have been analysed by real-time PCR assays. Results have led to a basic understanding of *Aj*5-HT4R in *A. japonicus*, and provide a foundation for further exploration of the cell signaling and regulatory functions of this receptor.

The biogenic amine serotonin (5-hydroxytryptamine; 5-HT), first isolated in 1948^1^, is a well-known neurotransmitter and neuromodulator. It has been described by Dr Barnes as the happy hormone^2^, involved in natural reward-related physiology and behaviour^3^, from feeding to sexual activity. Serotonin is biochemically derived from tryptophan and has various functions in different phyla, including both vertebrates and invertebrates, and plays a modulatory role in numerous physiological processes such as feed intake, reproduction, immunity and stress responses^4^,^5^,^6^.

Presently, seven classes of 5-HT receptors, 5-HT1 to 5-HT7, have been identified. Aside from 5-HT3, which is a ligand-gated ion channel that belongs to the nicotinic acetylcholine receptor superfamily^7^, the 5-HT receptors belong to the superfamily of G protein coupled receptors (GPCRs)^8^,^9^. Orthologous G protein-coupled 5-HT receptors have been identified and functionally characterised in deuterostomes including humans^10^, mammals^11^, birds^12^ and fishes^13^, and in protostomes including nematodes^14^, crustaceans^15^ and insects^16^,^17^. Furthermore, these G protein-coupled 5-HT receptors are mainly classified in three groups: (1) 5-HT1 and 5-HT5 receptors that couple preferentially to Gi proteins and inhibit cAMP synthesis, (2) 5-HT2 receptors that activate Gq proteins, which mediate the hydrolysis of inositol phosphates and cause a subsequent increase in cytosolic Ca^2+^, and (3) 5-HT4, 5-HT6, and 5-HT7 receptors that couple to Gs proteins and promote cAMP formation^18^,^19^. Additionally, an extra novel serotonin receptor (named Pr5-HT8) has been identified from larval *Pieris rapae* and, and represents the first recognized member of a novel 5-HT receptor class with a unique pharmacological profile^20^. In marine invertebrates or dormancy animals, only several serotonin receptors have been identified from the barnacle, *Balanus amphitrite* Darwin^21^ and the marine mollusc *Aplysia*^22^,^23^, however, the 5-HT receptors and their physiological functions in marine invertebrates still remain largely unknown. Meanwhile, aside from several gene expression analyses on 5-HT receptor-like genes^24^, our understanding of the molecular and pharmacological properties of 5-HT receptors in echinoderms is presently limited. The objective of this study was to identify the molecular and ligand activity characterisation of sea cucumber 5-HT4 receptor.

Primary identification and pharmacological characterisation of the 5-HT4 receptor was reported in 1988 through the activation of ligand 5-HT in cultured mouse embryonic collicular neurones^25^. Following this initial report, numerous selective 5-HT4 receptor agonists have been recognized that target 5-HT4 receptors in the central nervous system and peripheral organs and tissues^26^,^27^,^28^, and which thus have great therapeutic potential to treat patients suffering from a variety of diseases. In different peripheral organs, including the gastro intestinal (GI) tract, heart, and lung, 5-HT4 receptor activation is involved in various physiological processes^29^,^30^,^31^. For example, 5-HT4 receptor activation mediates peristaltic reflex activity due to its impaction on the enteric cholinergic axis^32^ and reversion of respiratory depression in etorphine-immobilisation^33^.

The sea cucumber *Apostichopus japonicus* (Selenka), is a temperate marine invertebrate animal in the family Stichopodidae whose growth is influenced by variation in water temperature^34^. When seawater temperature rises to a certain level during summer, most individuals of *A. japonicus* migrate to deeper water where they stop moving and feeding, and enter a dormant state known as aestivation. Aestivation of *A. japonicus* can last for as long as 4 months each year, and, in some regions of China, an aestivated *A. japonicus* can lose approximately 30–50% of its body mass^35^. During this inactive period, the rate of oxygen consumption is extremely depressed and associated with obvious hypometabolism^36^. Recent research, including transcriptomic, proteomic, and epigenetic, has characterised functional genes in active and aestivating *A. japonicus*, and has begun to reveal the mechanisms behind this process^37^,^38^,^39^,^40^,^41^, which has greatly advanced our understanding.

To our knowledge, little is currently known about the regulatory role of 5-HT4R in the extensive physiological adjustment during *A. japonicus* aestivation. In this present study, we report for the first time cloning and functional characterisation of a putative 5-HT4 receptor from the *A. japonicus*. Our results indicate that upon stimulation with 5-HT, the putative 5-HT4 receptor induces intracellular cAMP accumulation in dose-dependent manner and undergoes rapid internalisation mammalian cell line. Further investigation of the transcriptional expression profiles of this putative 5-HT4R between active and aestivated *A. japonicus* and phosphofructokinase (*Aj*PFK) in 5-HT administrated *A. japonicus* in three peripheral tissues (intestine, muscle and respiratory tree) indicates that *Aj*5-HT4R is potentially associated with respiratory depression in aestivated *A. japonicus*.

## Results

### Isolation and Characterisation of *Aj*5-HT4R cDNA

**Figure 1:**
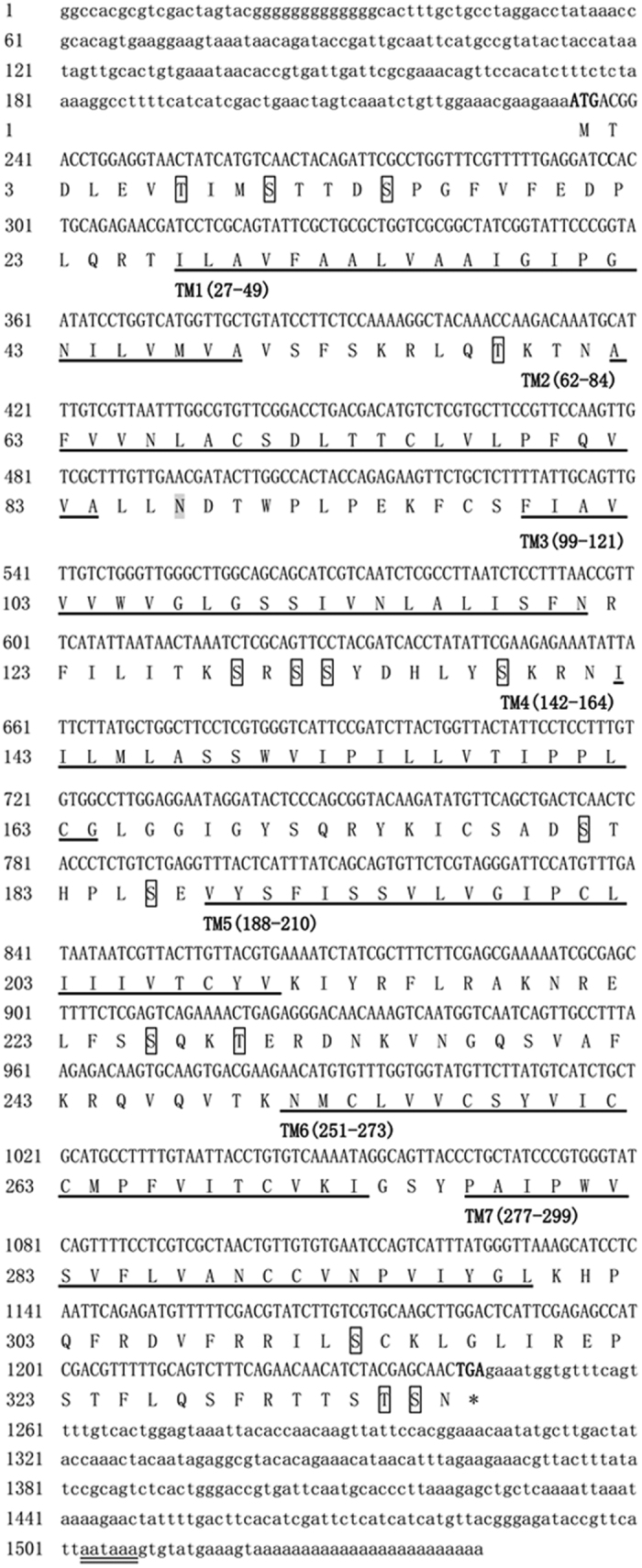
*Aj*5-HT4R cDNA sequence and deduced amino acid sequence. The seven transmembrane domains (TM1-TM7) are noted by the black underline. The N-glycosylated sites are highlighted in gray. The phosphorylation sites are labeled in box with full lines. The initiation codon (ATG) and the termination codon (TGA) are shown in bold. The potential polyadenylation signal (AATAAA) is noted by the double underscore. The numbers on the left refer to the position of the nucleotides and the amino acids.

The full-length 1,544 bp *Aj*5-HT4R cDNA sequence cloned from *A. japonicus* contains an open reading frame (ORF) 1,011 bp in length encoding 336 amino acids, a 5′ untranslated region (UTR) 233 bp in length, and 3′ UTR 300 bp in length (Fig. 1). The cDNA sequence was submitted to the NCBI GenBank under accession no. KX583229. One potential polyadenylation signal was identified within the 3′ UTR. The putative protein was predicted to have a molecular mass of 37.22 kDa and an isoelectric point (pI) of 9.29. The amino acid sequence of *Aj*5-HT4R contains the following potential sites: one typical N-glycosylation site (N87) within EC1, two conserved cysteine residues (C97 and C177) within EC1 and EC2, and 16 phosphorylation sites at 11 serine (S10, S14, S129, S131, S132, S138, S181, S186, S226, S313, and S333), four threonine (T7, T58, T229, and T332), and one tyrosine (Y133) residues (Fig. 1). The predication of 7tm_1 domain of this putative amino acid sequence indicated that *Aj*5-HT4R was a member of the rhodopsin-type (class A) GPCR family.

**Figure 2:**
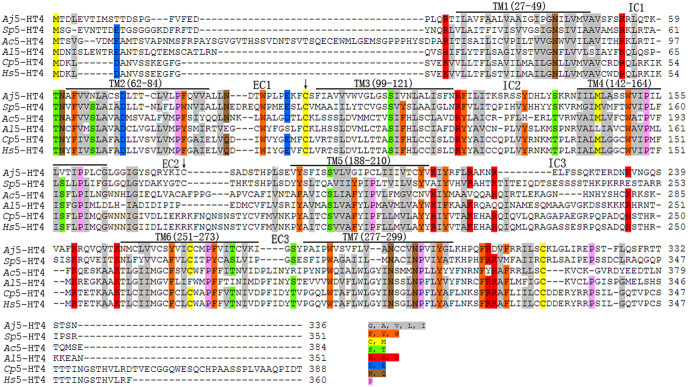
Alignment of the deduced *Aj*5-HT4R amino acid sequence with sequences from other species. Sequences of *Strongylocentrotus purpuratus* 5-HT4 receptor (*Sp*5-HT4R), *Aplysia californica* 5-HT4 receptor (*Ac*5-HT4R), *Austrofundulus limnaeus* 5-HT4 receptor (*Al*5-HT4R), *Cavia porcellus* 5-HT4 receptor (*Cp*5-HT4R) and *Homo sapiens* 5-HT4 receptor (*Hs*5-HT4R) were obtained from GenBank, along with a list of accession numbers (Suppl, Table S1). Alignment was generated using ClustalW and color align property was generated using Sequence Manipulation Suite online. The seven transmembrane domains (TM1–TM7) are marked with a black horizontal line above the sequence alignment. The three extracellular (EC) and three intracellular (IC) rings are noted above the sequence alignment. Black ↓ indicates the conserved cysteine residues. Percentage of sequences that must agree for identity or similarity coloring was set as 80%.

**Figure 3:**
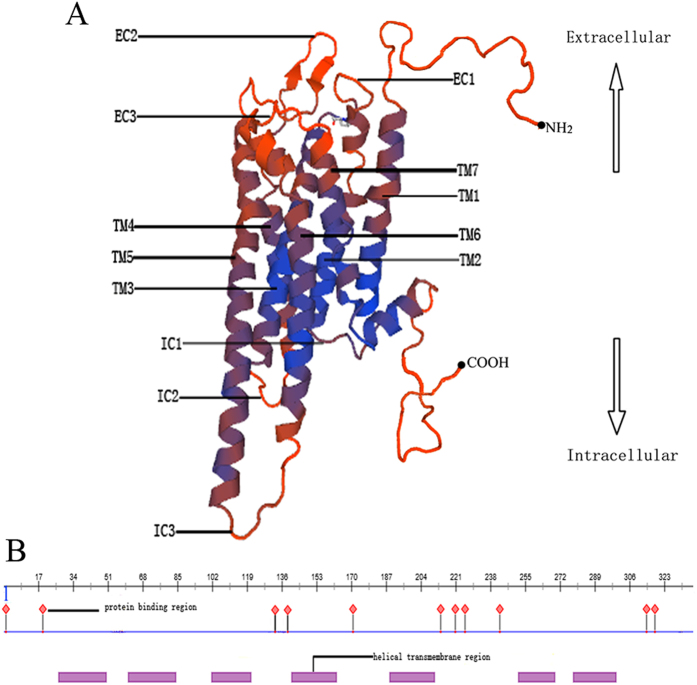
Predicted *Aj*5-HT4R protein structure and respective domain. (**A**) Predicted 3D structure of the *Aj*5-HT4R protein. Seven transmembrane domains (TM1-TM7), three extracellular (EC) rings and three intracellular (IC) rings are marked. The predicted 3D structure of the *Aj*5-HT4R protein was generated from SWISS-MODEL. (**B**) *Aj*5-HT4R protein binding domain and transmembrane region. The Dashboard overview was generated from PredictProtein.

The deduced amino acid sequence of *A. japonicus* 5-HT4R was compared with five other homologous 5-HT4R sequences, which vary in length from 351 to 388 amino acid residues. Pairwise ClustalW analysis of these amino acid sequences was carried out to evaluate homologous relationships. The predicted *A. japonicus* 5-HT4R amino acid sequence showed similarity to *Strongylocentrotus purpuratus* putative 5-HT4R sequence (40% identity), and other reported 5-HTR amino acid sequences, albeit with lower levels of similarity (from 22% to 27% identity). Multiple sequence alignment analysis revealed conservation in the 7tm_1 domain of HT4R sequences from various species including *A. japonicus* (Fig. 2). Apart from *A. japonicus* compared to *S. purpuratus* 5-HT4R, significant variation was observed in the length of C terminal regions (Fig. 2). Protein structure of *Aj*5-HT4R was predicted using SWISS-MODEL (Fig. 3A), whilst secondary structure was predicted by PredictProtein (Fig. 3B). Homology modelling revealed that this protein was similar to 4nc3.1.A in the Protein Data Bank. The protein binding region sites were predicted and marked on the constructed model.

**Figure 4:**
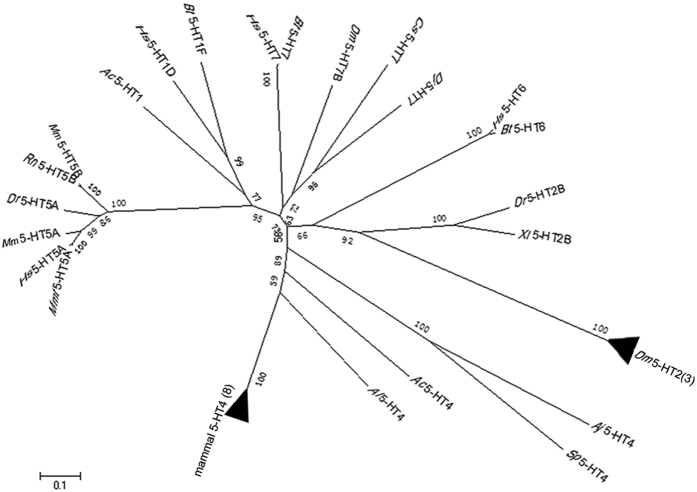
Phylogenetic tree based on amino acid of 5-HTRs. The tree was constructed based neighbor-joining algorithms using MEGA 6.0. The topological stability of the NJ tree was achieved by running 1000 bootstrapping replications. Bootstrap values (%) are indicated by numbers at the nodes. The numerical number in parentheses showed the number of sequences used in each taxon. The GenBank accession numbers and identities are listed in Suppl. Table S2.

To examine the relationship of *Aj*5-HT4R with 5-HTRs from various other species, a phylogenetic tree was constructed with Mega 6.0 using ClustalW multiple alignment and protein sequences of *Aj*5-HT4R and 32 alternate 5-HTRs that existed in the gene bank (Fig. 4). This revealed that 5-HTRs could be divided into six groups: 5-HT1, 5-HT2, 5-HT4, 5-HT5, 5-HT6, and 5-HT7 receptors. The deduced *Aj*5-HT4R protein sequence was positioned alongside 5-HT4 receptors in the phylogenetic tree, which, apart from the echinoderm 5-HT4R sequence, all grouped together.

### Cellular localisation and internalisation of *Aj*5-HT4R

**Figure 5:**
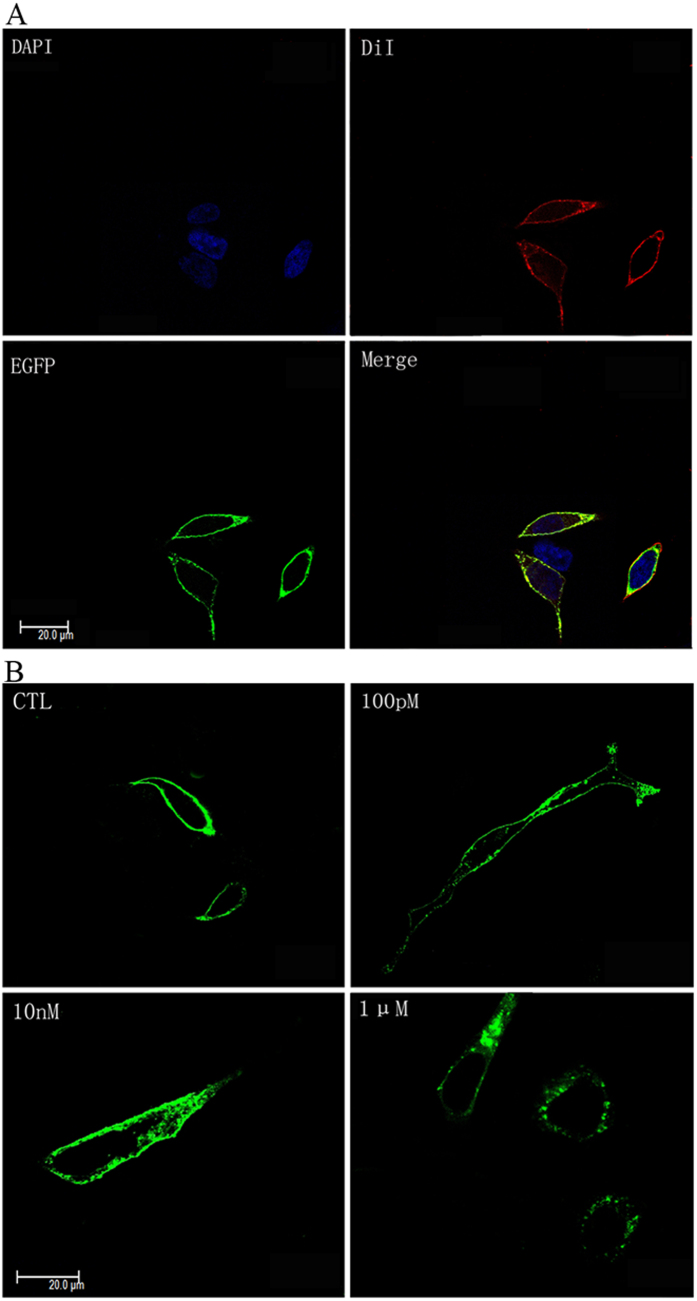
Confocal microscopy of HEK293 cells expressing the *Aj*5-HT4R-EGFP fusion protein. (**A**) *Aj*5-HT4R distribution in HEK293 cells. Cells were stained with a membrane plasma probe (DiI) and a nuclei probe (DAPI). Cells stably expressing *Aj*5-HT4R-EGFP were seeded on glass bottom six-well plates overnight, incubated with DiI (10 μM) and DAPI, and examined by confocal microscopy as described in the section Methods. (**B**) Internalisation of *Aj*5-HT4R in HEK293 cells. HEK293 cells transfected with *Aj*5-HT4R-EGFP were activated by treatment with 100 pM, 10 nM and 1 μM 5-HT during 60 min and detected by confocal microscopy. The “CTL” refers to control without 5-HT stimulation. All images represent at least three independent experiments.

To analyse the possible sub-cellular localisation of *Aj*5-HT4R, the *Aj*5-HT4R-EGFP vector was constructed which expressed *Aj*5-HT4R with enhanced green fluorescent protein (EGFP) fused to the C-terminus. HEK293 cells were transiently transfected with the *Aj*5-HT4R-EGFP vector and GFP signal was analysed by confocal microscopy. A high level of specific fluorescence was observed in a substantial proportion of transfected cells 48 h post transfection following a 24 h starvation period in serum-free medium. As expected, fluorescence was detected on cells transfected with the *Aj*5-HT4R-EGFP plasmid with significant cell surface expression apparent and minimal intracellular accumulation in the absence of 5-HT (Fig. 5A).

In order to visualise the internalisation and trafficking of *Aj*5-HT4R, HEK293 cells expressing *Aj*5-HT4R-EGFP were treated with varying concentrations of 5-HT. As described above, in the absence of 5-HT, fluorescence of *Aj*5-HT4R-EGFP was mainly localised in the plasma membraneand only marginally in intracellular vesicles (Fig. 5B). Following 1 μM 5-HT treatment, *Aj*5-HT4R-EGFP was rapidly and efficiently redistributed in the cytoplasm with distinct perinuclear accumulation (Fig. 5B), an effect that was not observed following treatment with 5-HT at lower concentrations (100 pM and 10 nM).

### cAMP accumulation in *Aj*5-HT4R expressing cells stimulated by 5-HT

**Figure 6:**
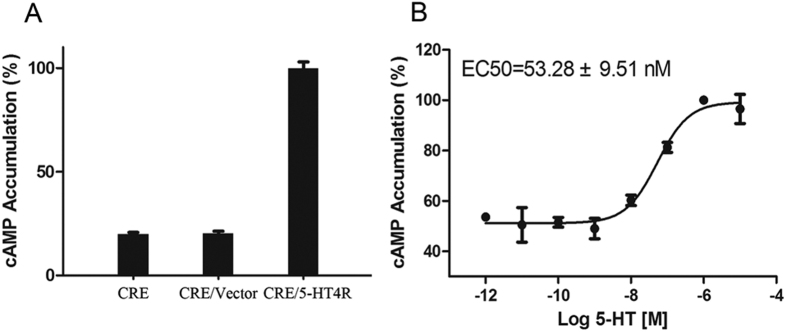
5-HT induced cAMP accumulation in HEK293 cells stably expressing Flag-*Aj*5-HT4R. (**A**) cAMP accumulation in HEK293 cells transientlyco-transfected with Flag-*Aj*5-HT4R and pCRE-Luc, and was determined in response to 5-HT treatment (1 μM). (**B**) cAMP accumulation in HEK293 cells stably expressing Flag-*Aj*5-HT4R/CRE-Luc was assayed in response to different doses of 5-HT. Data are expressed as the mean ± S.E. (n = 3).

cAMP accumulation depends upon the coupling of *Aj*5-HT4R to G proteins. Following 1 μM 5-HT treatment of HEK293 cells transfected with pCRE-Luc vector or co-transfected with pCRE-Luc and pCMV-Flag vectors, no change in cAMP level was detected. Conversely, cAMP levels were significantly elevated following a similar treatment of HEK293 cells co-transfected with Flag-*Aj*5-HT4R and pCRE-Luc (Fig. 6A). To evaluate the dose-response relationship between 5-HT and *Aj*5-HT4R in stimulating cAMP production, HEK293 cells co-transfected with Flag-*Aj*5-HT4R and pCRE-Luc were treated with different 5-HT concentrations, which showed that intracellular cAMP accumulated in a dose-dependent manner with an EC50 of 53.28 ± 9.51 nM (Fig. 6B).

### Expressional quantification of *Aj*5-HT4R mRNA and *Aj*PFK mRNA

**Figure 7:**
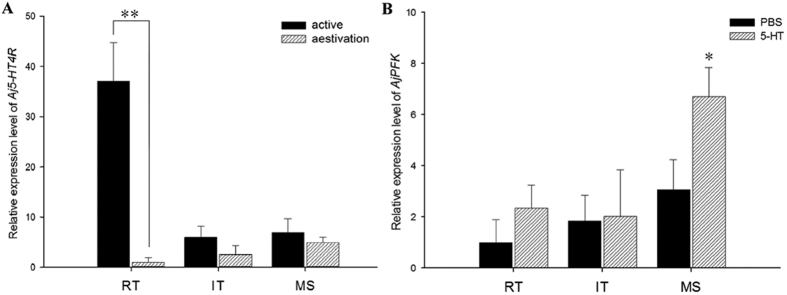
Relative expression of *Aj5-HT4R* and *AjPFK* in sea cucumbers. (**A**) Relative expression of *Aj5-HT4R* in different tissues of active and aestivating sea cucumbers. Total RNA was isolated and purified from the respiratory tree (RT), intestine (IT) and muscle (MS). The expression value was normalised against the expression of the internal control gene (*β-actin* and *β-tubulin*). Each symbol and verticalbar represents mean ± SD (n = 6). Double asterisk above the bars indicates extremely significant differences (*P* < 0.01) between active and aestivation. (**B**) Tanscriptional variation of *AjPFK* in different tissues of 5-HT administrated sea cucumbers. Total RNA was isolated and purified from the respiratory tree (RT), intestine (IT) and muscle (MS). The expression value was normalised against the expression of the internal control gene (*β-actin* and *β-tubulin*). Each symbol and verticalbar represents mean ± SD (n = 6). Asterisk above the bars indicates extremely significant differences (*P* < 0.05) between control (PBS) and experimental (5-HT) groups.

Real-time quantitative PCR assays were conducted to examine the expression patterns of *Aj5-HT4R* and *AjPFK* in various tissues of adult *A. japonicus*. Gene expression was normalised against expression of the house-keeping gene, *β-actin* (ACTB) and *β-tubulin* (TUBB). *Aj*5-HT4R mRNA was ubiquitously detected in respiratory tree, intestine, and muscle tissues of active and aestivated sea cucumbers. *Aj5-HT4R* expression was lower in intestine and muscle tissue during aestivation, although this difference was not statistically significant (Fig. 7A). An extremely significant decrease in *Aj5-HT4R* expression (*p* < 0.01), however, was observed in respiratory tree tissue (Fig. 7A). Moreover, to further evaluate the possible physiological role of *Aj5-HT4R* in the regulation of aestivation, the direct effect of 5-HT administration on the *Aj*PFK mRNA expression was investigated. 5-HT was administrated to the aestivated *A. japonicas* by intraperitoneal injection, and then real-time PCR was used to quantitatively analyse the transcriptional level of *Aj*PFK in respiratory tree, intestine, and muscle tissues. As shown in Fig. 7B, the experimental group with 5-HT administration exhibited a significant increase in *Aj*PFK mRNA in the muscle than that of the control group with vehicle (*p* < 0.05).

## Discussion

Serotonin (5-HT) produces its diverse effects through a variety of membrane-bound receptors in the central and peripheral system, and in the periphery such as the gut, cardiovascular system and blood^42^,^43^. 5-HT receptors are well-documented in vertebrates, especially in humans^44^. However, 5-HT receptors in marine invertebrates are comparably less characterised. In order to obtain further insight into serotonergic signaling and its functions in the echinoderm *A. japonicus*, we identified and functionally characterised the 5-HT4 receptor in this organism.

The putative 5-HT4R cDNA sequence cloned from *A. japonicus* encodes a 336 amino acid mature protein. The mature protein contains two conserved cysteine residues, a potential N-linked glycosylation site and 16 potential phosphorylation sites, which are thought to be involved in the regulation of protein trafficking and localisation, function and diversity, and signal transduction^45^,^46^. The alignment of *Aj*5-HT4R protein sequence and other species showed that *Aj*5-HT4R has the highest identity (40%) to the putative 5-HT4R of *S. purpuratus*. This is consistent with the evolutionary pattern of these two species. Moreover, sequence analysis revealed that the putative *Aj*5-HT4R together with its orthologs from other species contain amino acid motif NPxxY located at the end of the seventh transmembrane domain, which is a highly conserved among GPCRs; while unlike most of the rhodopsin-type (class A) GPCRs, the putative *Aj*5-HT4R and *Sp*5-HT4R contain a variant of the consensus D/E-R-Y/F motif with the replacement of aspartate residue with asparagines. The D/E-R-Y/F motif located between the TM3 and the second intracellular loop is a highly conserved motif in the majority of the class A GPCRs. Previous studies have indicated that both negatively charged residue D/E and positively charged residue R of the D/E-R-Y/F are involved in governing receptor conformation and G protein coupling/recognition^47^. The murine cytomegalovirus (MCMV) gene M33 and rat cytomegalovirus (RCMV) gene R33 encode proteins homologous to cellular chemokine receptors^48^, and both contain a variant NRY motif^49^. Studies using mutational analysis have demonstrated that the D/E residue of the D/E-R-Y/F motif is involved in agonist-dependent and independent activation of receptor response^50^,^51^,^52^,^53^. However, more efforts are required for elucidation of the function of the NRF motif of *Aj*5-HT4R.

Phylogenetic analysis was carried out to investigate the evolutionary relationship between 5-HTRs except for 5-HT3R which belonged to ligand-gated ion channel. The resulting phylogenetic tree revealed segmentation into six main groups (Fig. 4). Two groups comprise of receptors coupled to Gi proteins (5-HT1 and 5-HT5 receptors), three groups comprise of receptors coupled to Gs proteins (5-HT4, 5-HT6 and 5-HT7 receptors) and one group comprises of receptors coupled to Gq proteins (5-HT2 receptors) which is close to the 5-HT6 receptors group. The *Aj*5-HT4R clustered together with the putative 5-HT4R from *S. purpuratus* and was located distant to vertebrate 5-HT4Rs. Also, interestingly, the protostomian 5-HT4R from *Aplysia californica*, whose function in protein kinase C (PKC) activation was established by experimental data^23^, is also clustered in the 5-HT4R group and even closer to vertebrate 5-HT4Rs compared to echinoderm 5-HT4Rs. This suggests a common ancestor for 5-HT4Rs in deuterostomia and protostomia. Nevertheless, further experiments are required to establish the functional character and associated cell signalling pathways of echinoderm 5-HT4Rs and discover the potential difference underlying the evolutionary distance between echinoderm and vertebrate 5-HT4Rs.

In the current study, we used HEK293 cells as a heterologous expression system to functionally characterise *Aj*5-HT4R. We have demonstrated, for the first time, that *Aj*5-HT4R is activated by 5-HT with EC50 value of the response in the nanomolar range. Our data provide evidence that *Aj*5-HT4R is a Gs protein-coupled receptor, specifically activating adenylyl cyclase to induce intracellular cAMP formation. This is in high agreement with previous studies^18^,^19^. Additionally, to further assess *Aj*5-HT4R functional activity as a transmembrane receptor, fusion expression of *Aj*5-HT4R with the enhanced green fluorescent protein (EGFP) at the C-terminus was used to further assess *Aj*5-HT4R functional activity. Significant cell surface expression was observed under fluorescence microscopy (Fig. 5A), suggesting that C-terminal EGFP fusion did not affect *Aj*5-HT4R expression and transportation. Upon activation by agonist, fluorescence of *Aj*5-HT4R-EGFP was rapidly and efficiently internalised from cell surface into the cytoplasm. Agonist-induced internalisation is a well-known phenomenon for most GPCRs that control GPCR signalling, ensuring appropriate cellular responses to stimuli^54^. Our observation confirmed that *Aj*5-HT4R is a complete and functional receptor.

5-HT4 receptors are widely expressed in the body, including the central nervous system and peripheral tissues, and they exert pleiotropic effects after being activated by 5-HT. The activation of neuronal 5-HT4 receptors results in a facilitation of neurotransmitter release in the brain and the periphery. Clues to the possible functions of the 5-HT4 recptors might be obtained from its tissue distribution. In brain, 5-HT4 receptors reside in the limbic system executing their memory and learning effects^55^, and in hippocampus enhancing cognition and neuroprotection^18^. In the enteric nervous system, 5-HT4 receptor activation stimulates gastrointestinal motility in the GI tract^56^,^57^. In respiratory system, 5-HT4 receptor mediates respiratory efforts on stimulate breathing in human lung tissue^31^,^58^, and is related with pulmonary function in mice^59^. To investigate the potential regulatory functions of 5-HT4 receptor in *A. japonicus*, the expression profile of *Aj5-HT4R* in respiratory tree, intestine and muscle tissues of active and aestivated *A. japonicus* was quantitively analysed in the current study. Results indicated that the *Aj5-HT4R* transcript was highly expressed in respiratory tree. By contrast, we found *Aj5-HT4R* mRNA levels to be low in sample from all three tissues of aestivated individuals, and the lowest expression was detected in respiratory tree tissue. This is consistent with the observation of the respiratory depression of *A. japonicus* during aestivation^36^,^60^, it suggests that the reduction of *Aj5-HT4R* expression is likely related to the respiratory depression of *A. japonicus* during aestivation. In addition, we also demonstrated that intraperitoneal administration of 5-HT resulted in a significant increase of *Aj*PFK expression. PFK is a rate limiting enzyme of glycolysis, and plays a key regulatory role in metabolic regulation^61^. It has been demonstrated that 5-HT involved in glucose uptake in skeletal muscle and regulate the glycolytic metabolism^62^. Although our data derived from *AjPFK* expression analysis do not exclude the possible roles of other 5-HT receptors in the modulation of the *A. japonicas* aestivation, together with the observation of *Aj5-HT4R* expression pattern in respiratory tree, it suggests that 5-HT/*Aj*5-HT4R system is likely involved in the metabolic regulation of the *A. japonicas* aestivation. However, more efforts are required to clarify its functional role of *Aj5-HT4R* in the regulation of the respiratory depression.

In conclusion, the full-length *Aj*5-HT4R cDNA sequence has been identified and its resulting protein has been functionally characterised in the present study. The transmembrane nature of *Aj*5-HT4R has been demonstrated, and internalisation of this receptor was activated by direct interaction with 5-HT. Intracellular cAMP accumulation in response to 5-HT treatment ina dose-dependent manner has been verified. The transcriptional decrease of *Aj5-HT4R* in respiratory treeduring aestivating periods suggests the potential relation between 5-HT4R and respiratory depression of *A. japonicus* during aestivation. On the other aspect, the preliminary estimated pharmacological profile of 5-HT in aestivating *A. japonicus* indicates the functional activity of 5-HT in metabolic regulation and suggests the potential invlovement of 5-HT receptors on respiratory control. The results presented here lead to a basic understanding of 5-HT4R in *A. japonicus* and further experiments should be conducted to clarify associated signalling pathways and the physiological function of this receptor.

## Methods

### Sample Collection

For cDNA cloning and *Aj5-HT4R* expression analysis in various tissues, adult individuals of the sea cucumber *A. japonicus* were collected separately from culture ponds in Qingdao (Shandong, China in April and June 2016). Each batch was acclimated in seawater aquaria (salinity range: 32.41–34.37). Individuals in the active group (79.3 ± 5.1 g body mass) were kept at a constant temperature (16.0 ± 0.5 °C) and fed with a formulated diet (45% marine mud, 50% Sargasso, and 5% shrimp shell powder). Individuals in the aestivating group (68.9 ± 3.2 g body mass) were also kept at a constant temperature (25.0 ± 0.5 °C) to maintain aestivation. After 15 days, respiratory tree, intestine and muscle tissues were sampled from six individuals for both groups and stored in liquid nitrogen for future use. For the experiment of adding 5-HT in aestivated sea cucumbers, intraperitoneal injection with 5 mM 5-HT (1 μL/g body weight) was carried out every 24 hours (PBS injection for control), and their respiratory tree, intestine and muscle tissues were sampled 15 days later for gene expressional quantification of phosphofructokinase (*AjPFK*, accession number KT779933) in glycolytic metabolism pathway.

### Preparation of cDNA and Rapid amplification of cDNA ends (RACE)

Total RNA was isolated from intestine, respiration tree and muscle tissue of *A. japonicus* using TRIzol reagent (TaKaRa, Kusatsu, Japan) and phenol chloroform. The integrity of the total RNA was verified by electrophoresis, and RNA concentration and quality were determined using a Nanodrop 2000 (Thermo Fisher Scientific).

10 μg and 1 μg total intestine RNA was obtained to conduct 5′RLM-RACE and 3′RLM-RACE protocols, respectively. These were conducted using a FirstChoice^®^ RLM-RACE kit (Ambion Inc., TX, USA) following manufacturer’s instructions, and samples were then stored at −20 °C for the subsequent PCR step.

For each sample, 1 μg total RNA was reverse transcribed into single-stranded cDNA by incubating with M-MLV reverse transcriptase and oligo(dT)20 (Promega Inc., Shanghai, China) at 42 °C for 1 h. An RNase inhibitor (Promega Inc., Shanghai, China) was used during cDNA synthesis to avoid RNA degradation. The cDNA was kept at −20 °C for real-time PCR. The gene-specific primers (listed in Table 1) were designed according to the partial coding sequence of *Aj5-HT4R* from the expressed sequence tag (EST) library, which was constructed by Trinity RNA-Seq Assembly using seven pubilshed transcriptome databases (SRR1185973, SRR1139215, SRR414930, SRR414929, SRR414928, SRR414927, SRR414926).

### Sequences characterisation and phylogenetic relationships

The *Aj*5-HT4R cDNA sequence was used to query known sequences in GenBank using the blastx utility, BLASTX 2.2.29+ (http://blast.ncbi.nlm.nih.gov/). The full length cDNA sequence of *A. japonicus* 5-HT4 receptor was translated into the predicted amino acid sequence with DNAMAN 8.0. Physicochemical properties of proteins depended on Protparam (http://www.expasy.org/tools/protparam.html). N-glycosylation and phosphorylation sites were located with the NetNGlyc1.0 Server (http://www.cbs.dtu.dk/services/NetNGlyc/) and NetPhos 2.0 Server (http://www.cbs.dtu.dk/services/NetPhos/), respectively. Analysis of transmembrane in the protein was achieved by Tmpred (http://www.ch.embnet.org/software/TMPRED_form.html). Protein domains were predicted with InterProScan software (http://www.ebi.ac.uk/interpro/search/sequence-search) and SMART (http://smart.embl-heidelberg.de/). Analysis of secondary structure was predicted with PredictProtein (http://www.predictprotein.org/). The deduced amino acid sequences were aligned using ClustalW. Color Align Property was generated by Sequence Manipulation Suite (http://www.bioinformatics.org/sms2/color_align_prop.html). *Aj*5-HT4R protein structure was predicted using SWISS-MODEL (http://swissmodel.expasy.org/). Phylogenetic tree construction was based on the Neighbor-Joining (NJ) Method of Molecular Evolutionary Genetics Analysis (MEGA 6.0). The bootstrap value was repeated 1000 times to obtain the confidence value for the analysis.

### Construction of the Mammalian Expression Vectors

To construct the *Aj*5-HT4R plasmid, reverse transcript PCR (RT-PCR) was performed as described in method of “Preparation of cDNA”. To amplify the CDS of *Aj5-HT4R*, forward primer (*Aj*5-HT4R-vec-F) and reverse primers (*Aj*5-HT4R-vec-R-EGFP) and (*Aj*5-HT4R-vec-R-Flag) were designed based on the full-length cDNA sequence and to allow for subcloning into the pEGFP-N1 and pCMV-Flag plasmids, respectively (Table 1). The pEGFP-N1 and pCMV-Flag vectors were purchased from Clontech Laboratories, Inc. (Palo Alto, CA), and Sigma (St.Louis, MO), respectively. The PCR products were inserted into the final pEGFP-N1 and pCMV-Flag expression vectors using the EcoRI and KpnI restriction enzymes (Beyotime, Haimen, China) and Rapid DNA Ligation Kit (Beyotime, Haimen, China). The constructed vectors were named *Aj*5-HT4R-EGFP and Flag-*Aj*5-HT4R, respectively, and sequenced to verify sequence fidelity, orientation, and reading frame.

### Transfection

The human embryonic kidney cell line (HEK293) was maintained in Dulbecco’s modified Eagle’s medium (DMEM, Hyclone, Logan, UT, USA) supplemented with 10% foetal bovine serum (FBS, HyClone, Logan, UT, USA) and 4 mM L-glutamine (Invitrogen, Madison, WI, USA). The *Aj*5-HT4R-EGFP and Flag-*Aj*5-HT4R vectors were transfected into HEK293 cells using Lipofectamine 2000 (Invitrogen, Madison, WI, USA) according to manufacturer’s instructions.

### Internalisation assay and Confocal Microscopy

HEK293 cells expressing *Aj*5-HT4R-EGFP were seeded onto glass coverslips coated with 0.1 mg/ml of poly-L-lysine and allowed to attach overnight under normal growth conditions. After 24 h, the HEK293 cells starved for a further 24 h in serum-free medium to eliminate the effects of endogenous 5-HT in FBS. For receptor surface expression analysis, cells were stained with the membrane probe DiI (Beyotime, Haimen, China) at 37 °C for 5–10 min, fixed with 4% paraformaldehyde for 15 min, and finally incubated with DAPI (Beyotime, Haimen, China) for 10 min. For the internalisation assay, cells expressing *Aj*5-HT4R-EGFP were treated with 100 pM, 10 nM and 1 μM 5-HT (sigma, Saint Louis, USA) for 60 min at 37 °C, then fixed with 4% paraformaldehyde for 15 min. Cells were visualized by fluorescence microscopy on a Leica TCS SP5II laser scanning confocal microscope using a HCX PL APO lambda blue 63× 1.4 oil immersion lens.

### cAMP accumulation measurement

After seeding in a 96-well plate overnight, HEK293 cells stably cotransfected with Flag-*Aj*5-HT4R and pCRE-Luc vectors were grown to 80–85% confluence, before being starved for 24 h in serum-free medium to eliminate the effects of the endogenous 5-HT in FBS. Cells were thentreated with the indicated concentration of 5-HT(1 pM, 10 pM, 100 pM, 1 nM, 10 nM, 100 nM, 1 mM, and 10 mM) in DMEM without FBS and incubated for 4 h at 37 °C, with application repeated a total of three times. Luciferase activity was detected using a firefly luciferase assay kit (Kenreal, Shanghai, China).

### Real-time quantitative PCR (qRT-PCR)

For qRT-PCR, *β-actin* (ACTB) and *β-tubulin* (TUBB) were chosen as the internal control (housekeeping) genes and gene-specific primers were designed based on the ORF sequences^63^,^64^. Specific qRT-PCR primers for *Aj5-HT4R* and *AjPFK* were designed based on CDS (Table 1). The primers were tested to ensure amplification of single discrete bands with no primer-dimers. qRT-PCR assays were carried out using the SYBR PrimeScript™ RT reagent Kit (TaKaRa, Kusatsu, Japan) following manufacturer’s instructions, and ABI 7500 Software v2.0.6 (Applied Biosystems, UK). qRT-PCR was performed for 35 cycles with the following condition: 95 °C/5 s, 60 °C/30 s. The relative level of gene expression was calculated using the 2^−△△Ct^ method and data was normalised by geometric averaging of the internal control genes^65^,^66^. Differences between experimental and control groups were tested using one-way analysis of variance (ANOVA) followed by Tukey’s post hoc test, using PASW Statistics 18.00 (SPSS Inc., Chicago, IL, USA). Significance was set at *P* < 0.05, and extremely significance was set at *P* < 0.01.

## Additional Information

**How to cite this article**: Wang, T. *et al*. Identification and functional characterisation of 5-HT4 receptor in sea cucumber *Apostichopus japonicus* (Selenka). *Sci. Rep.* **7**, 40247; doi: 10.1038/srep40247 (2017).

**Publisher's note:** Springer Nature remains neutral with regard to jurisdictional claims in published maps and institutional affiliations.

## Supplementary Material

Supplementary Tables

## Figures and Tables

**Table 1:** Sequence of primers used for 5′ and 3′RACE of *A. japonicus* 5-HT4 receptor and qPCR analysis for*Aj5-HT4R*.

Primer	Sequence (5′-3′)	Application
5′-outer	TGATAGTGGCCAAGTATCGTTC	*Aj5-HT4R* 5′ RACE
5′-inner	GTCAGGTCCGAACACGCCAA	
3′-outer	TGCTGGCTTCCTCGTGGGTCATTCC	*Aj5-HT4R* 3′ RACE
3′-inner	CTCAACTCACCCTCTGTCTGAGGTT	
*Aj*5-HT4R-vec-F	GGAATTCATGACGGACCTGGAGGTAAC	Vectorsconstruction
*Aj*5-HT4R-vec-R-EGFP	GGGGTACCAAGTTGCTCGTAGATGT	*Aj*5-HT4R-EGFP construction
*Aj*5-HT4R-vec-R-Flag	GGGGTACCTCAGTTGCTCGTAGATGT	Flag-*Aj*5-HT4R construction
*Aj5-HT4R*-q-F	TTCAGCTGACTCAACTCACCCTCTG	*Aj5-HT4R*qPCR
*Aj5-HT4R*-q-R	CTGATTGACCATTGACTTTGTTGTC	
*AjPFK*-q-F	AAAGAAGCTTTGTGATGGAGGTC	*AjPFK* qPCR
*AjPFK*-q-R	TTTTATCTTCCCAATCATCAGCA	
TUBB-F	CACCACGTGGACTCAAAATG	Internal control
TUBB-R	GAAAGCCTTACGACGGAACA	
ACTB-F	AAGGTTATGCTCTTCCTCACGC	Internal control
ACTB-R	GATGTCACGGACGATTTCACG	
