# Supplementary material for: Identification and functional characterisation of 5-HT4 receptor in sea cucumber Apostichopus japonicus (Selenka)
Source: Sci Rep. 2017 Jan 6;7:40247. doi: 10.1038/srep40247 (PMC5216381; doi:10.1038/srep40247)
Supplement: Supplementary Tables [file srep40247-s1.doc]

**Identification and functional characterisation of 5-HT4 receptor in sea cucumber *Apostichopus japonicus* (Selenka)**

Tianming Wang^1^, Zhen Yang^1^, Naiming Zhou^2^, Lina Sun^3^, Zhenming Lv^1^, Changwen Wu^1^

^1^National Engineering Research Center of Marine Facilities Aquaculture, Marine Science College, Zhejiang Ocean University, Zhoushan, Zhejiang 316022, People’s Republic of China

^2^Institute of Biochemistry, College of Life Sciences, Zijingang Campus, Zhejiang University, Hangzhou, Zhejiang 310058, People’s Republic of China

^3^Key Laboratory of Marine Ecology and Environmental Sciences, Institute of Oceanology, Chinese Academy of Sciences, Qingdao, Shandong 266071, People’s Republic of China

Correspondence and requests for materials should be addressed to T.W. (wtmzjuedu@163.com, wangtianming@zjou.edu.cn) or N.Z. (znm2000@yahoo.com)

**Supplementary Table S1.** List of 5-HT4Rs sequences, and their respective sources, used to alignment analysis. Accession numbers and identities are shown. Accession numbers were taken from GenBank and identities were computed using Bioedit.

| Receptor | Species | | Accession | | Identities (%) | |
| --- | --- | --- | --- | --- | --- | --- |
| *Aj*5-HT4 | | *Apostichopus japonicus* | KX583229 | 100 | |  |
| *Sp*5-HT4 | *Strongylocentrotus purpuratus* | | XP_003727696.1 | | 40 | |
| *Ac*5-HT4 | *Aplysia californica* | | AEA76357.1 | | 27 | |
| *Al*5-HT4 | *Austrofundulus limnaeus* | | XP_013863315.1 | | 22 | |
| *Cp*5-HT4 | *Cavia porcellus* | | NP_001166434.1 | | 26 | |
| *Hs*5-HT4 | *Homo sapiens* | | CAA73109.1 | | 26 | |

**Supplementary Table S2.** List of 5-HTRs sequences, and their respective sources, used to generate the phylogenetic tree. Accession numbers and identities are shown. Accession numbers were taken from GenBank and identities were computed using Bioedit.

| Receptor | Species | Accession | Identities (%) |
| --- | --- | --- | --- |
| *Aj*5-HT4 | *Apostichopus japonicus* | KX583229 | 100 |
| *Ac*5-HT1 | *Aplysia californica* | AAC28786.1 | 29 |
| *Hs*5-HT1D | *Homo sapiens* | AAA60316.1 | 20 |
| *Bt*5-HT1F | *Bos taurus* | DAA33629.1 | 21 |
| *Dr*5-HT2B | *Danio rerio* | AAI62703.1 | 24 |
| *Xl*5-HT2B | *Xenopus laevis* | CAD71264.1 | 23 |
| *Dm*5-HT2B D | *Drosophila melanogaster* | NP_649806.2 | 26 |
| *Dm*5-HT2B E | *Drosophila melanogaster* | NP_001262373.1 | 26 |
| *Dm*5-HT2B F | *Drosophila melanogaster* | AHN57237.1 | 26 |
| *Sp*5-HT4 | *Strongylocentrotus purpuratus* | XP_003727696.1 | 40 |
| *Ac*5-HT4 | *Aplysia californica* | AEA76357.1 | 27 |
| *Al*5-HT4 | *Austrofundulus limnaeus* | XP_013863315.1 | 22 |
| *Ss*5-HT4 | *Sus scrofa* | NP_001001267.1 | 24 |
| *Bt*5-HT4 | *Bos taurus* | NP_001035575.1 | 24 |
| *Hs*5-HT4 | *Homo sapiens* | CAA71462.1 | 26 |
| *Cp*5-HT4 | *Cavia porcellus* | NP_001166434.1 | 26 |
| *Rn*5-HT4 c | *Rattus norvegicus* | EDM14630.1 | 25 |
| *Rn*5-HT4 e | *Rattus norvegicus* | EDM14632.1 | 25 |
| *Mm*5-HT4 a | *Mus musculus* | EDL09741.1 | 25 |
| *Mm*5-HT4 d | *Mus musculus* | EDL09744.1 | 25 |
| *Mmt* 5-HT5A | *Macaca mulatta* | NP_001182753.1 | 24 |
| *Hs*5-HT5A | *Homo sapiens* | CAA57168.1 | 25 |
| *Mm*5-HT5A | *Mus musculus* | EDL37224.1 | 22 |
| *Dr*5-HT5A | *Danio rerio* | NP_001119882.2 | 23 |
| *Rn*5-HT5B  *Mm*5-HT5B | *Rattus norvegicuss*  *Mus musculus* | NP_077371.1  NP_034613.2 | 23  23 |
| *Hs*5-HT6 | *Homo sapiens* | AAA92622.1 | 21 |
| *Bt*5-HT6 | *Bos taurus* | NP_001192646.1 | 21 |
| *Hs*5-HT7 | *Homo sapiens* | AAC37538.1 | 21 |
| *Bt*5-HT7  *Dm*5-HT7B | *Bos taurus*  *Drosophila melanogaster* | DAA14954.1  AGB96510.1 | 21  23 |
| *Cs*5-HT7 | *Clonorchis sinensis* | GAA50114.1 | 27 |
| *Dj*5-HT7 | *Dugesia japonica* | BAI44327.1 | 30 |
